# Abdominal pain patterns during COVID-19: an observational study

**DOI:** 10.1038/s41598-022-18753-0

**Published:** 2022-08-29

**Authors:** Alexandre Balaphas, Kyriaki Gkoufa, Nicola Colucci, Konstantinos-Cédric Perdikis, Christophe Gaudet-Blavignac, Zoltan Pataky, Sebastian Carballo, Frédéric Ris, Jérôme Stirnemann, Christian Lovis, Nicolas Goossens, Christian Toso

**Affiliations:** 1 Division of Digestive Surgery, University Hospitals of Geneva, Geneva, Switzerland; 2 Department of Surgery, University of Geneva, Geneva, Switzerland; 3 Division of Endocrinology, Diabetology, Nutrition and Patients’ Education, University Hospitals of Geneva, Geneva, Switzerland; 4 Department of Clinical-Surgical, Diagnostic and Pediatric Sciences, University of Pavia, Pavia, Italy; 5 Division of General Internal Medicine, University Hospitals of Geneva, Geneva, Switzerland; 6 Division of Medical Information Sciences, University Hospitals of Geneva, Geneva, Switzerland; 7 Division of Gastroenterology and Hepatology, University Hospitals of Geneva, Geneva, Switzerland

**Keywords:** Digestive signs and symptoms, Gastrointestinal diseases, Infectious diseases, Viral infection

## Abstract

Abdominal pain and liver injury have been frequently reported during coronavirus disease-2019 (COVID-19). Our aim was to investigate characteristics of abdominal pain in COVID-19 patients and their association with disease severity and liver injury.

Data of all COVID-19 patients hospitalized during the first wave in one hospital were retrieved. Patients admitted exclusively for other pathologies and/or recovered from COVID-19, as well as pregnant women were excluded. Patients whose abdominal pain was related to alternative diagnosis were also excluded.

Among the 1026 included patients, 200 (19.5%) exhibited spontaneous abdominal pain and 165 (16.2%) after abdomen palpation. Spontaneous pain was most frequently localized in the epigastric (42.7%) and right upper quadrant (25.5%) regions. Tenderness in the right upper region was associated with severe COVID-19 (hospital mortality and/or admission to intensive/intermediate care unit) with an adjusted odds ratio of 2.81 (95% CI 1.27–6.21, p = 0.010). Patients with history of lower abdomen pain experimented less frequently dyspnea compared to patients with history of upper abdominal pain (25.8 versus 63.0%, p < 0.001). Baseline transaminases elevation was associated with history of pain in epigastric and right upper region and AST elevation was strongly associated with severe COVID-19 with an odds ratio of 16.03 (95% CI 1.95–131.63 p = 0.010).

More than one fifth of patients admitted for COVID-19 presented abdominal pain. Those with pain located in the upper abdomen were more at risk of dyspnea, demonstrated more altered transaminases, and presented a higher risk of adverse outcomes.

## Introduction

Gastrointestinal symptoms have been identified amid most common extra pulmonary manifestations of 2019-coronavirus disease (COVID-19)^1^. Notably, abdominal pain has been observed during severe acute respiratory syndrome-coronavirus 2 (SARS-CoV2) infections with a prevalence ranging from 1.9 to 14.5%^2^. In a meta-analysis on more than 6000 patients, Mao et al. demonstrated that the presence of abdominal pain was associated with severe forms of COVID-19 with an odd ratio of 7.10^3^. Hepatocellular injury pattern, of which the hallmark is an elevation of liver function tests (LFT), was also related to severe COVID-19^3^. COVID-19 liver injury has mostly been seen in 16 to 53% hospitalized patients and includes mild elevation of LFT, mostly of aspartate aminotransferase (AST), acute hepatitis and even acute cholecystitis^1,4–6^.

It has not been elucidated yet whether abdominal pain and elevation of LFT are just bystanders and indicators of critical illness or if they represent distinct clinical patterns of COVID-19. Moreover, management of patients with acute abdomen in the setting of a SARS-CoV2 systemic infection becomes challenging for the clinician as it can mislead diagnosis.

The aim of this retrospective study was to clarify patterns of abdominal pain related to SARS-CoV2 infection during the first wave of the pandemic and their relations with severe COVID-19 clinical outcomes, modification of LFT and other clinical features.

## Patients and methods

The project was approved by the Geneva ethics committee on research (CCER 2020-01141). The need for consent was waived by the ethics committee. However, patients who have signed University Hospitals of Geneva’s general consent and were opposed to health data utilization were excluded. Patients over 16 years old admitted in the University Hospitals of Geneva with a positive SARS-CoV2 PCR from the beginning of the first COVID-19 wave in Switzerland, i.e. from 25.02.2020 until 30.06.2020, were identified from electronic patient file records. All patients corresponding to our criteria were included. Patients were included if they were admitted for COVID-19, for COVID-19 and one or more other diagnosis, or if they were tested positive for SARS-CoV2 during an ongoing hospitalization as COVID-19 could have influenced their hospital course. Asymptomatic patients admitted for other medical conditions with SARS-CoV2 PCR positivity at the time of admission or previously to admission, were excluded. Patients with resolved COVID-19 and pregnant women were also excluded. Polymerase chain reaction (PCR) detection of SARS-CoV2 RNA was performed either in the Geneva University Hospitals or in private laboratory using different kits.

Data were retrieved using both automatized informatics methods (at the end of July 2020) for laboratory values and outcome variables and manually (for abdominal pain-related qualitative variables, history of surgery, surgery, dyspnea, smoking and alcohol consumption, from August to December 2020), by a team of four investigators (AB, KG, NC, KCP). Pain was not quantified with numeric rating scale (NRS) of pain as its report was too variable in patients’ records. Primary outcome was defined by either intermediate care unit admission (IMCU) during the same hospitalization, to intensive care unit admission (ICU) during the same hospitalization or to overall hospital mortality. Criteria for IMCU admission were blood oxygen saturation < 90% despite Fi0_2_ > 50% with Venturi Mask without signs of respiratory distress. In MCU, patient alternated between high flow oxygen (HFO) and Continuous Positive Airway Pressure (CPAP). If blood oxygen saturation remained < 90% despite Fi0_2_ > 80% with HFO/CPAP or if patients presented signs of respiratory distress, they were transferred to ICU for oro-tracheal intubation and mechanical ventilation. Non-invasive ventilation was not used for COVID-19 patients with acute respiratory insufficiency, but in the recovery phase after being extubated. Chest CT was not used to assess COVID-19 severity, but only if the suspicion of a secondary diagnostic was raised (e.g. pulmonary embolism). Overall hospital mortality was examined at a minimum follow-up of 30 days after inclusion and maximum of 3 months. Any abdominal pain since COVID-19 onset until 30 days after admission, not explained by a clear alternative diagnostic, was considered as being caused by COVID-19. Body mass index (BMI), alcohol consumption, age, sex, dyspnea, and abdominal surgery during hospitalization were identified as potential confounding factors. Other aims focused on abdominal pain patterns, association between LFT and abdominal pain and COVID-19 severity. To describe the demography of the cohort, other variables such as previous abdominal surgeries and smoking status were also collected. Ambiguities regarding pain or other variables were adjudicated with a team discussion twice a month during the data collection process. Data were verified by AB and KP and secondary and independently by NG. Data were processed and recoded using R (R Core Team, 2020) and further analyzed with Stata 15 (StataCorp, College station, TX, USA).

Complete case analysis was performed according to the extent of missing observations, variable type (outcome or cofounding factor) and its risk of missing not at random, otherwise variables were excluded of analysis when the risk to introduce bias was too high. For some adjustment categorized variables, secondary sensibility analyses were performed with the imputation of a separate category for missing variables. All variables were first graphically represented to assess their distributions. When appropriate, mean or median were used. Two-sided statistic tests were ran using the appropriate test (Chi^2^ or Fisher exact). A p value < 0.05 was considered as statistically significant. For all logistic regression models homoscedasticities of continuous variables were checked and when necessary, variables were categorized. Goodness of fit of each model was assessed with the Hosmer–Lemeshow test.

## Results

### Demographic characteristics and fate of hospitalized patients with SARS-CoV2 infection

**Figure 1:**
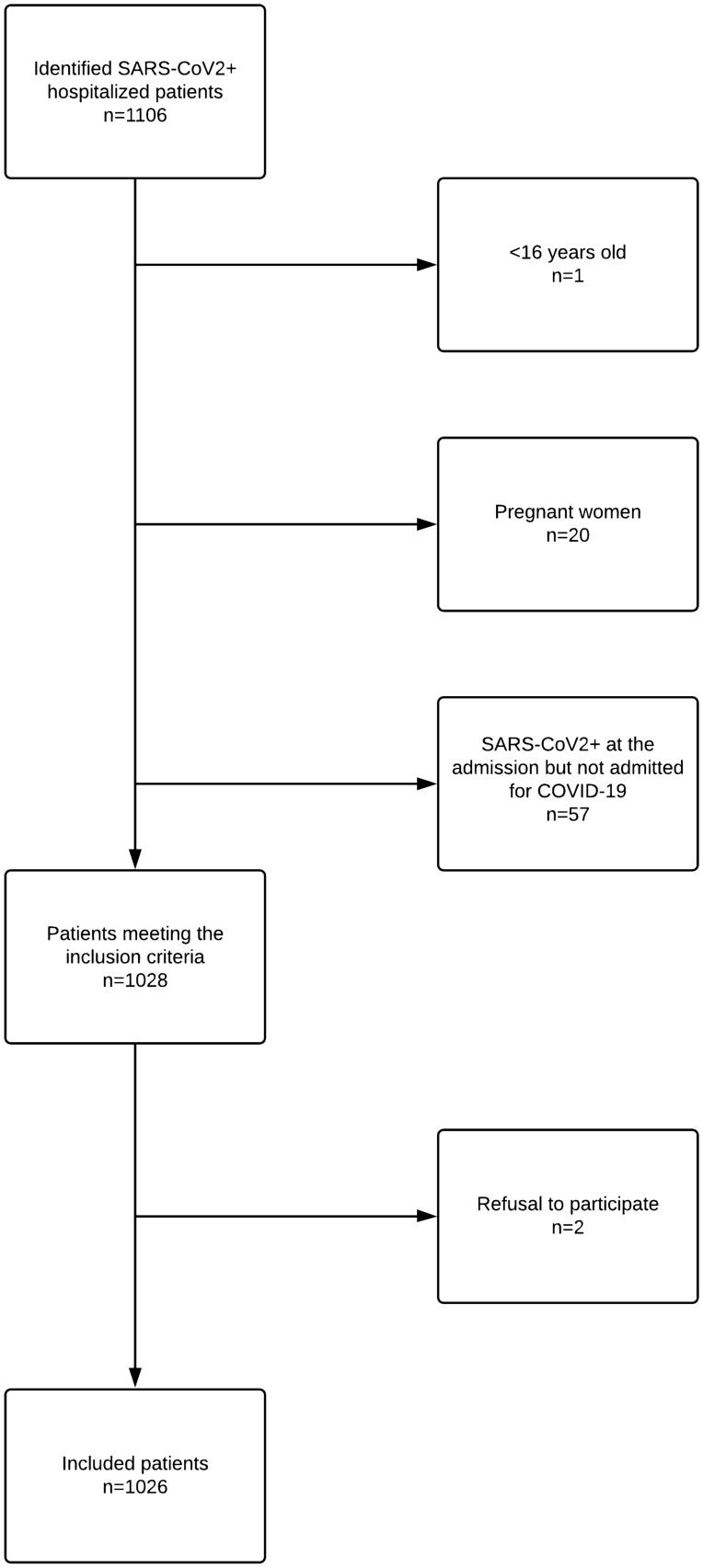
Workflow of the inclusion process.

A total of 1106 patients were identified, of which 1026 were included (Fig. 1). The complete demography of the cohort is summarized in Table 1. The majority (62.9%) of admitted patients were admitted/transferred in standard acute care division whereas 20.4% were admitted/transferred to the geriatric division and 43% finally transferred to rehabilitation unit. During their hospitalization, 24 patients underwent surgical procedure, including 12 abdominal surgeries. SARS-CoV2 infection led to intermediate care unit admission of 17.4% patients of the cohort and to intensive care unit of 13.3% patients (for invasive mechanical ventilation), whereas hospital mortality reached 17.2% (Table 2).

**Table 1:** Demographic variables of the included patient.

		*n*
**Age at admission median (range) [years]**	69.7 [16–101]	1024
**Female (%)**	466 (45.6)	1024
**Male n (%)**	555 (54.4)	1024
**Smoking n (%)**		
No smoking	522 (56.9)	918
Actively smoking	90 (9.8)	918
Former smoking	306 (33.3)	918
**Alcohol consumption n (%)**		
High risk	79 (9.4)	844
Former high risk	45 (5.3)	844
Low risk	368 (43.6)	844
No alcohol consumption	352 (41.7)	844
BMI mean ± sd [kg/m^2^]	25.6 ± 6.3	272
**Categorized BMI n (%) [kg/m**^**2**^**]**		
< 18	26 (9.6)	272
18–25	116 (42.7)	272
25–30	69 (25.4)	272
30–35	39 (14.3)	272
35–40	16 (5.9)	272
≥ 40	6 (2.2)	272
**History of abdominal surgery n (%, CI)**		
Bariatric surgery	11 (1.1, 0.5–1,9)	1026
Cholecystectomy	94 (9.2, 4,5–11.1)	1026
Partial hepatectomy	4 (0.4, 0.1–1)	1026
Other liver surgeries	6 (0.6, 0.2–1.3)	1026

The third column represents the number of valid (nonmissing) observations. High risk alcohol consumption: ≥ 21 unit/week for men or ≥ 14/week for women. Low risk alcohol consumption: < 21 unit/week for men or < 14/week for women. BMI: body mass index.

**Table 2:** Outcome variables.

Evolution of SARS-CoV2 infection	*n* (%, 95% CI)
**Mortality|ICU admission|IMCU admission**	355/1026 (34.6, 31.7–37.6)
Mortality	176/1021 (17.2, 15–19.7)
ICU admission	135/1018 (13.26, 11.2–15.5)
IMCU admission	178/1026 17.35, (15.1–19.8)

Presence and characteristics of abdominal pain during SARS-CoV2 infection	*n* (%, 95% CI)
**History of abdominal pain**	200/1026 (19.5, 17.1–22.1)
Diffuse abdominal pain	82/192 (42.7)
Localized abdominal pain	110/192 (57.3)
**Abdominal point tenderness**	165/1020 (16.2, 14.0–18.6)
Diffuse abdominal point tenderness	42/165 (24.9)
Localized abdominal point tenderness	127/165 (75.2)
History of abdominal pain or abdominal point tenderness	243/1026 (23.7, 21.1–26.4)
Murphy’s sign	14/1010 (1.4, 0.07–2.3)
**Other outcome variables**	
Total hospital stay median(range) [days]	9.6 (1–107)
Surgery during COVID-19 n (%, CI)	24 (2.34, 1.5–3.5)
Cholecystectomy n (%)	4/24 (16.7)
Appendicectomy n (%)	3/24 (12.5)
Other abdominal procedure n (%)	5/24 (20.8)

Primary outcome was defined by either ICU admission, MCU admission or death during the hospitalization (whatever the origin) Among 1026 patients, 200 had a history of abdominal pain whereas 165 out of 1020 (missing data for 6) reported pain induced by abdomen palpation. In 8 out of 200 patients with history of abdominal pain, localization was missing (192 patients with known localization). ICU: intensive care unit, IMCU: intermediate care unit.

### Pattern of COVID-19 related abdominal pain

**Figure 2:**
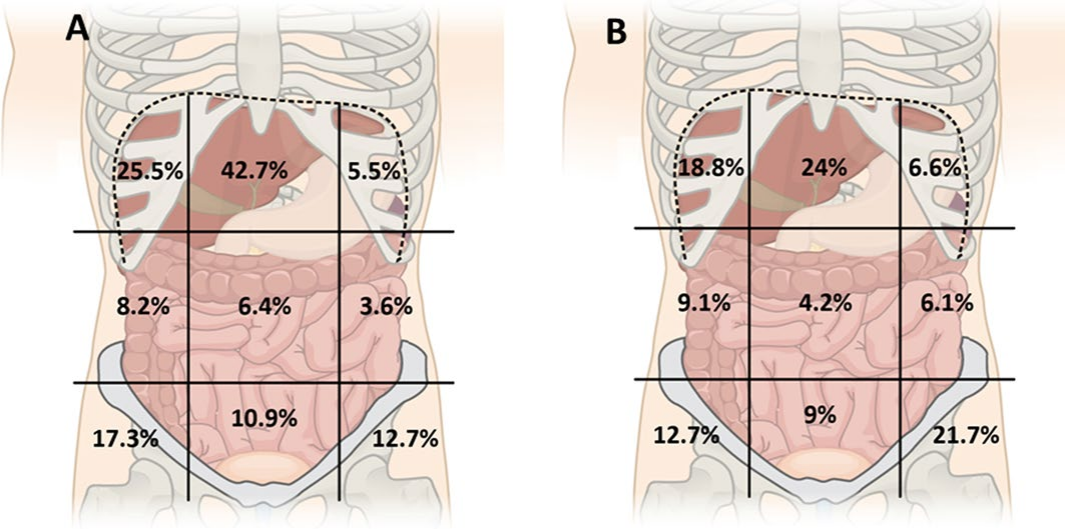
Localization of abdominal pain. (**A**) abdominal pain on history. (**B**) abdominal point tenderness (each percentages represented the frequency of pain in the concerned region independently from the other localizations). (Adapted from Abdominal Quadrant Regions Cleaned, Jmarchn. Creative Commons Attribution-Share Alike 3.0 Unported).

Amongst included patients, 19.5% (17.1–22.1%) reported a history of abdominal pain, which was diffuse in 82 out of 192 (42.7%) patients (localization of pain was missing in 8 patients) and well localized in 110 out of 192 (57.3%) patients (Table 2). After physical examination 16.2% (14.0–18.6%) patients had abdominal point tenderness which was well localized in 75.2% of cases. Overall, 243 patients had either history of abdominal pain or abdominal pain elicited by physical examination. Focusing on localized abdominal pain, we demonstrated that patients reported more frequently spontaneous pain in the upper abdominal regions: epigastric (42.7%) or right hypochondria (25.5%) (Fig. 2). This tendency was consistent when looking at abdominal point tenderness localization frequencies.

### Relation between abdominal pain and COVID-19 outcomes

Association between abdominal pain and more severe forms of COVID-19 defined by intermediate care unit admission, intensive care unit admission and/or hospital mortality was studied with univariate and multivariate logistic regression (Table 3). Right upper region tenderness or the presence of Murphy’s sign were independent predictors of severe COVID-19 outcomes. When adjusting for abdominal surgery during hospital stay, dyspnea, sex and age which were considered as confounding factors, odds ratios of severe COVID-19 reached respectively 2.81 (1.27–6.21, p = 0.010) and 6.06 (1.70–21.60, p = 0.005) for right upper region tenderness and Murphy’s sign. Due to the presence of missing data, categorized BMI and categorized alcohol consumption were not included in the primary analysis and were considered in an additional sensibility analysis (Table S1). Interestingly, epigastric region tenderness adjusted for these two additional confounding factors was inversely correlated with severe COVID-19 outcomes with an odd ratio of 0.38 (0.15–0.96, p = 0.042).

**Table 3:** Mortality and/or intensive care unit admission and/or intermediate care unit admission association with pattern of abdominal pain before and after adjustment for sex, age, abdominal surgery during hospital stay and dyspnea (complete case analyses).

	Univariate logistic regression			Multivariate logistic regression		
	OR	95% CI	p value	OR	95% CI	p value
History of abdominal pain or abdominal point tenderness	1.02	0.76–1.38	0.887	1.24	0.89–1.73	0.201
History of abdominal pain	0.97	0.70–1.34	0.842	1.16	0.81–1.66	0.410
Abdominal point tenderness	1.20	0.85–1.69	0.306	1.42	0.97–2.07	0.074
History of right upper region pain	1.23	0.57–2.66	0.598	1.27	0.54–2.97	0.580
History of epigastric region pain	0.64	0.33–1.24	0.184	0.92	0.45–1.86	0.812
Right upper region tenderness	2.06	1.01–4.23	**0.047**	2.81	1.27–6.21	**0.010**
Epigastric region tenderness	0.32	0.13–0.77	**0.011**	0.42	0.17–1.05	0.064
Murphy’s sign	4.80	1.52–15.70	**0.008**	6.06	1.70–21.60	**0.005**

OR: odds ratio, CI: confidence interval.

Significant values are given in bold.

### Relation between level of transaminases and abdominal pain pattern or severe COVID-19 outcomes

A cut-off of 5× the upper level of transaminases was considered indicative of a viral infection-associated hepatitis. Considering the baseline value (i.e. first value after or at admission), we demonstrated with univariate and adjusted multivariate analysis (adjustment for sex, age, and abdominal surgery during hospital stay) that history of right upper region pain, epigastric region pain and Murphy’s sign were associated with 5× the upper level of transaminases elevation (Table 4). Abdominal point tenderness in other abdominal regions did not demonstrate an association with elevation of transaminases, whereas the association with Murphy’s sign was strong regarding AST elevation with an odds ratio of 24.54 (4.18–144.12, p < 0.001). Moreover, univariate or multivariate logistic regression analysis demonstrated that baseline elevation of 5× the upper level of AST predict severe COVID-19 outcomes with an odds ratio of 16.03 (1.95–131.63, p = 0.010) after adjustment for sex and age. Sensitivity analysis adding categorized body mass index (BMI) and categorized alcohol consumption did not induced major modifications in the strength of association and the p value (data not shown).

**Table 4:** Association between elevation of transaminases and abdominal pain pattern in SARS-CoV2 patients, before and after adjustment for sex, age, abdominal surgery during hospital stay (complete case analysis) and association between mortality and/or ICU admission and/or IMCU admission and elevation of transaminases before and after adjustment for sex, age, (complete case analyses).

Elevation of transaminases and abdominal pain pattern	Univariate logistic regression			Multivariate logistic regression		
	OR	95% CI	p value	OR	95% CI	p value
**History of abdominal pain or abdominal point tenderness**						
Baseline AST ≥ 5× upper value	0.71	0.15–3.34	0.668	0.85	0.18–4-03	0.841
Baseline ALT ≥ 5× upper value	2.85	0.37–21.93	0.315	1.66	0.16–17.25	0.669
**History of right upper region pain**						
Baseline AST ≥ 5× upper value	3.40	0.41–27.81	0.255	4.10*	0.48–34.84*	0.197*
Baseline ALT ≥ 5× upper value	11.63	1.16–116-78	**0.037**	30.94*	1.77–539.64*	0.019*
**History of epigastric region pain**						
Baseline AST ≥ 5× upper value	2.49	0.31–20.15	0.393	3.29	0.39–27.77	0.275
Baseline ALT ≥ 5× upper value	26.62	3.61–196.41	**0.001**	19.32	2.13–175.17	**0.008**
**Murphy’s sign**						
Baseline AST ≥ 5× upper value	16.81	3.17–89.09	**0.001**	24.54	4.18–144.12	** < 0.001**
Baseline ALT ≥ 5× upper value	23.30	2.23–243.75	**0.009**	17.77	0.92–343.41	0.057
**Mortality and/or ICU admission and/or IMCU admission and elevation of transaminases**						
Baseline AST ≥ 5× upper value	17.63	2.24–138.50	**0.006**	16.03	1.95–131.63	**0.010**
Baseline ALT ≥ 5× upper value	0.56	0.06–5.41	0.616	0.64	0.05–7.54	0.724

Patterns of abdominal point tenderness did not reach the level of significance and are not shown. OR: odds ratio, CI: confidence interval.

Significant values are given in bold.

*Model not valid.

### Relation between abdominal pain and dyspnea

In 98 patients out of 1026, abdominal pain was not associated with dyspnea (9.6%). Using multivariate analysis we demonstrated that patient with history of hypogastric and right and left lower region pain had less dyspnea. (Table 5) History of abdominal pain pattern was then recoded into upper (at least pain in one of the upper abdominal regions) or lower abdominal region (at least pain in one of the lower abdominal regions) variables with elimination of pain pattern in other regions which were rare according to the distribution frequency of abdominal pain (Fig. 2). Using these new variables, we studied their association with dyspnea using Cochrane’s Q test and demonstrated that 63% of patients with upper regions abdominal pain had dyspnea whereas only 25.8% of patients with lower regions had a history of dyspnea, this difference being statistically significant (Table S2).

**Table 5:** Association between dyspnea and abdominal pain pattern in SARS-CoV2 patients, before and after adjustment for sex, age, abdominal surgery during hospital stay (complete case analysis).

	Univariate logistic regression			Multivariate logistic regression		
	OR	95% CI	p value	OR	95% CI	p value
History of abdominal pain or abdominal point tenderness	0.88	0.66–1.19	0.415	0.92	0.68–1.25	0.597
History of abdominal pain	0.98	0.71–1.35	0.899	1.02	0.73–1.42	0.916
Abdominal point tenderness	0.70	0.50–0.98	0.038	0.75	0.53–1.07	0.112
History of right upper region pain	1.12	0.51–2.43	0.791	1.27	0.56–2.89	0.572
History of epigastric region pain	0.75	0.42–1.36	0.344	0.71	0.39–1.32	0.275
History of right lower region pain	0.21	0.77–0.60	**0.003**	0.22	0.08–0.63	**0.005**
History of hypogastric region pain	0.12	0.03–0.55	**0.006**	0.13	0.03–0.61	**0.010**
History of left lower region pain	0.10	0.02–0.45	**0.003**	0.10	0.02–0.48	**0.004**
Right upper region tenderness	0.97	0.47–2.03	0.944	1.06	1.06–2.28	0.887
Epigastric region tenderness	0.60	0.32–1.14	0.118	0.56	0.29–1.07	0.077
Right lower region tenderness	0.68	0.28–1.60	0.368	0.80	0.33–1.93	0.618
Hypogastric region tenderness	0.28	0.19–1.48	0.229	0.58	0.2–1.67	0.315
Left lower region tenderness	0.54	0.28–1.05	0.069	0.60	0.29–1.13	0.105

Each row presented the output of a separate model.

OR: odds ratio, CI: confidence interval.

Significant values are given in bold.

We also investigated the association between admission low PaO_2_/FiO_2_ and severe COVID-19 outcomes. Using the threshold of 40 kPa (300 mmHg) proposed by the Berlin consensus for Acute Respiratory Distress Syndrome (ARDS)^7^ and multivariate logistic regression, admission low PaO_2_/FiO_2_ was associated with severe COVID-19 outcomes but not with an increase mortality risk (Table S3). A combination of admission low PaO_2_/FiO_2_ and abdominal pain did not increase the association with severe COVID-19 outcomes and on the contrary, the Odds ratio was lower (5.43 versus 13.14). This can be explained by the bias that patients arriving in the emergency department with respiratory distress were not fit enough to report a full medical history and to have a complete head-to-toe assessment. Report of abdominal pain in these cases may have been overlooked.

## Discussion

In the present study, the prevalence of patients experiencing abdominal pain during COVID-19 was higher than in the reported literature (23.7 versus 14.5%)^8^. This may be explained by the fact that abdominal pain was broadly captured manually from medical records from COVID-19 symptoms onset and also throughout the entire hospital course. This strategy was adopted because the time course of this symptom remains unclear. Contrary to other studies, both history and physical examination was considered. Although this strategy may overestimate abdominal pain, particular care was taken to eliminate patients with abdominal pain from other evident etiologies (eg. coprostasis, gallbladder disease, etc.) and adjusted our analysis for abdominal surgery. As a quality control of the recording process, we calculated that median time between pain symptoms and SARS-CoV2 infection diagnosis was of 0 (interquartile range 7) days, indicating that a majority of abdominal pain were captured early during SARS-CoV2 infection.

In this work we unveiled the patterns of COVID-19-related abdominal pain which was never done before, to the best of our knowledge. Patients demonstrated the highest frequency of spontaneous or elicited pain in the upper abdomen with predominance of the epigastric and right upper regions. However, 17.1% of patient had a history of right lower region and 21.7% abdominal point tenderness in left lower region. We hypothesized that these two features could be the hallmark of a manifestation of COVID-19 that could involve small gut and/or colon instead of the liver. Unfortunately, as we did not collected data about transit, we do not know if this pattern of abdominal pain was preferentially associated with diarrheas, nausea or vomiting.

Using these pain patterns in our analysis, we then demonstrated an association between Murphy’s sign and right upper region tenderness and severe COVID-19 outcomes. Adjustment was based on supposed confusion factors but did not dramatically change the strength or the significance of the association. This primary analysis was limited by the possibility to adjust for BMI and alcohol consumption due to missing data and possible other unknown confounding factors. Therefore, we performed a secondary analysis as a sensibility analysis of the addition of BMI and alcohol consumption in the model (Table S1). In this secondary analysis, the associations mentioned earlier did not change. However, epigastric point tenderness which presented an inverse association with the composite outcome and was not statistically significant anymore after adjustment in the primary analysis, was again statistically significant with our secondary analysis. The p value of this association in this secondary analysis was close to 0.05 and this finding could be a type I error. Moreover, biliary tract or liver pain can both be felt in right hypochondria and epigastric region but tenderness is more precisely localized in right upper region after percussion, palpation or Murphy’s sign testing. Indeed, localized epigastric tenderness in COVID-19 might be related to gastritis and not to an injury to the liver or biliary tract. Of note, history of abdominal pain or tenderness in other regions of the abdomen and diffuse pain were not associated with severe COVID-19 outcomes.

Association between 5× the upper level of transaminases (250 UI) and abdominal pain was first studied using a two-way table at different time points (baseline transaminases, maximal value during the first 7 days or maximal value during the first 30 days after admission) (data not shown). This analysis only showed an association with the maximal value of transaminases during the first 30 days. As many factors can raise LFT during hospital course (for which we could not adjust), we decided to only consider the baseline value of transaminases and to run a logistic regression to adjust for confounding factors. Thus, we demonstrated different associations with AST and ALT and history of upper region pain but not with any pattern of abdominal point tenderness excepting Murphy’s sign.

Indeed, it has been demonstrated that SARS-CoV2 induced both an alternation of hepatic blood flow, as a systemic response to the infection, with sinusoid thrombi and a direct replication of virus in liver tissue^6^. These two features could indicate two distinct physio-pathological processes (a systemic reaction with activation of coagulation and a direct insult to the liver). Angiotensin-converting enzyme 2 (ACE2) receptors, which allow the entry of the SARS-CoV2 in cells, are prominent in liver tissue, particularly on bile duct cells^9^ but the virus can replicate in both hepatocytes^10,11^ and bile duct cells^12^. This could partially explain the elevation of LFT observed during COVID-19 while cytolytic pattern of injury is most frequent than cholestasic^13^. Elevation of LFT is multifactorial in COVID-19^6^ and in the present study, we stay focused on baseline LFT using stringent cut-off of 5× the upper level of transaminases (the hallmark of viral hepatitis^14^) to decrease the probability of considering drug-induced cytolysis. On the other hand, we excluded patients with COVID-19-induced mild elevation of transaminases which has been frequently reported^1^. However, while some authors suggest a distinction between COVID-19-induced hepatitis and mild cytolysis, our analysis did not demonstrate a multimodal but an unimodal and left-skewed distribution of transaminases, an observation already reported^15^.

While physiopathology of SARS-CoV2 infection on liver tissue has been investigated, little is known about the effects of the virus on the gut and its role on abdominal pain ^2^. The virus could directly replicate in epithelial and glandular cells at various localizations along the intestinal tract^16,17^ and it is supposed that gastrointestinal symptoms of COVID-19, including abdominal pain are induced by gut viral infection^1^. However, abdominal pain during COVID-19 might also be induced by hepatitis or an inflammation of biliary tract and we previously detected the presence of SARS-CoV2 RNA in gallbladder tissue from a patient with acalculous cholecystitis^5^. In the present study, the majority of patients described pain in upper abdominal regions, but a significant proportion also had a pain pattern localized in the lower abdomen. This could indicate that abdominal pain during COVID-19 is either induced by liver injury or gut infection.

**Figure 3:**
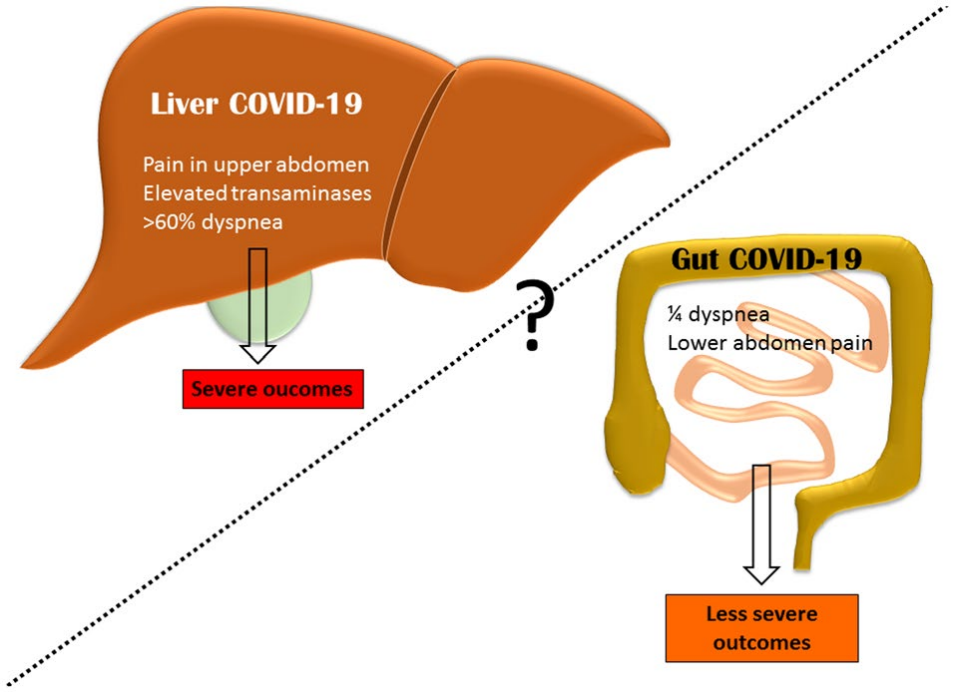
Proposed hypothesis of two forms of COVID-19 gastrointestinal symptoms: the liver and the gut COVID-19. Liver COVID-19 could be associated with upper abdomen pain and, dyspnea and more severe clinical outcomes while gut COVID-19 with lower abdomen pain and less severe outcomes.

In order to assess the hypothesis that COVID-19 induces two different manifestations on gastrointestinal system with opposite outcomes, we finally compared the localization of abdominal pain and the presence of dyspnea in a post hoc analysis. Using paired analysis, we showed that dyspnea was more prominent in patients with pain in the upper abdomen. According to our findings we built the hypothesis that two forms of COVID-19 may exist and could be characterized by different gastrointestinal manifestations and different outcomes (Fig. 3). The first form or liver-COVID-19 could be identified by elevated transaminases, abdominal pain localized preferentially in right upper region, dyspnea and a higher probability to be admitted to IMCU, ICU or to die during the hospitalization. The second form or gut-COVID-19 could be hallmarked by gastric and lower hemi-abdomen pain and could be associated with less severe outcomes regarding IMCU, ICU admission or death and less dyspnea.

The present work was limited by its retrospective design and the number of missing data for some variables. Data were missing due to a lack of accuracy of medical records but also because of research method limitations. These missing data had considerable consequences and we were not able to analyze other LFT such as alkaline phosphatase, bilirubin and γ-glutamyltransferase. Moreover, gastrointestinal symptoms are not limited to abdominal pain. Anorexia, diarrhea, nausea and/or vomiting as well as gastrointestinal bleeding have been frequently identified during COVID-19^1^. We deliberately choose not to look for them in order to reduce the number of variables we were manually looking for. Indeed, as the study was concentrating on abdominal pain and liver injury, we did not expect during protocol preparation that other gastrointestinal symptoms were of interest. Gut-COVID-19 might be associated with other gastrointestinal symptoms. Collecting observational studies in a meta-analysis, Mao et al. demonstrated that only patients with abdominal pain had severe form of COVID-19 whereas other gastrointestinal symptoms such as diarrhea, vomiting or nausea were not associated with severe outcomes^3^. Moreover, the frequent utilization of antibiotic and antiviral therapy (Hydroxychloroquine, ritonavir) could be a cause of gastrointestinal symptoms and a potential cofounding factor for this study as patient with severe COVID-19 could have been preferentially selected for experimental therapies^18^. However, experimental treatments were introduced in our hospital, only in severe COVID-19 patients, most of whom were already intubated in the ICU, making unlikely the report of abdominal pain. Also, other causes of abdominal pain could have falsely suggested an associated with COVID-19 such as diaphragmatic irritation or enteral nutrition, but we estimated that their effect is limited. Considering that pain in left upper region was rarely reported both during history and physical examination (respectively 5.5% and 6.6% cf. Fig. 2) it is unlikely that COVID-19 pneumopathy have induced exclusively right-sided extra-digestive abdominal pain, sparing left side. The effect of enteral nutrition was also negligible, as abdominal pain appeared early in the disease course (as reported earlier) and enteral nutrition was mainly limited to ICU patients with invasive mechanical ventilation unable to precisely report pain. Thus, this project deserves further studies with prospective design to verify our findings and hypotheses.

## Conclusion

More than one fifth of patients admitted for COVID-19 presented abdominal pain. Those with pain located in the upper abdomen were more at risk of dyspnea, demonstrated more altered transaminases, and presented a higher risk of adverse outcomes (death, ICU or IMCU admission). Further prospective study recording the exact pattern of abdominal pain and other abdominal symptoms (such nausea, vomiting and diarrhea) are mandatory to draw final conclusions.

## Supplementary Information

Supplementary Figure S1.

Supplementary Table S1.

Supplementary Table S2.

Supplementary Table S3.

Supplementary Table S4.

## Data Availability

The datasets generated and analyzed during the current study are not publicly available due their inclusion in a larger database owned by the Geneva University Hospitals but are available from the corresponding author on reasonable request.

## Abbreviations

ACE2: Angiotensin-converting enzyme 2
AST: Aspartate aminotransferase
ALT: Alanine aminotransferase
BMI: Body mass index
COVID-19: 2019-Coronavirus disease
LFT: Liver function tests
SARS-CoV-2: Severe acute respiratory syndrome-coronavirus 2

## References

[1] Gupta A (2020). Extrapulmonary manifestations of COVID-19. Nat. Med..

[2] Weng L-M, Su X, Wang X-Q (2021). Pain symptoms in patients with coronavirus disease (COVID-19): A literature review. J. Pain Res..

[3] Mao R (2020). Manifestations and prognosis of gastrointestinal and liver involvement in patients with COVID-19: A systematic review and meta-analysis. Lancet Gastroenterol. Hepatol..

[4] Scutari R (2020). Long-term SARS-CoV-2 infection associated with viral dissemination in different body fluids including bile in two patients with acute cholecystitis. Life.

[5] Balaphas A (2020). COVID-19 can mimic acute cholecystitis and is associated with the presence of viral RNA in the gallbladder wall. J. Hepatol..

[6] Cichoż-Lach H, Michalak A (2021). Liver injury in the era of COVID-19. World J. Gastroenterol..

[7] The ARDS Definition Task Force* (2012). Acute respiratory distress syndrome: The Berlin Definition. JAMA.

[8] Redd WD (2020). Prevalence and characteristics of gastrointestinal symptoms in patients with severe acute respiratory syndrome coronavirus 2 infection in the United States: A multicenter cohort study. Gastroenterology.

[9] Gavriatopoulou M (2020). Organ-specific manifestations of COVID-19 infection. Clin. Exp. Med..

[10] Lagana SM (2020). Hepatic pathology in patients dying of COVID-19: A series of 40 cases including clinical, histologic, and virologic data. Mod. Pathol..

[11] Wang Y (2020). SARS-CoV-2 infection of the liver directly contributes to hepatic impairment in patients with COVID-19. J. Hepatol..

[12] Zhao B (2020). Recapitulation of SARS-CoV-2 infection and cholangiocyte damage with human liver ductal organoids. Protein Cell.

[13] Metawea MI, Yousif WI, Moheb I (2021). COVID 19 and liver: An A-Z literature review. Dig. Liver Dis..

[14] Giannini EG, Testa R, Savarino V (2005). Liver enzyme alteration: A guide for clinicians. CMAJ.

[15] Lei F (2020). Longitudinal association between markers of liver injury and mortality in COVID-19 in China. Hepatology.

[16] Xiao F (2020). Evidence for gastrointestinal infection of SARS-CoV-2. Gastroenterology.

[17] Lamers MM (2020). SARS-CoV-2 productively infects human gut enterocytes. Science.

[18] Alberca GGF, Solis-Castro RL, Solis-Castro ME, Alberca RW (2021). Coronavirus disease-2019 and the intestinal tract: An overview. World J. Gastroenterol..

