# Supplementary material for: Abdominal pain patterns during COVID-19: an observational study
Source: Sci Rep. 2022 Aug 29;12:14677. doi: 10.1038/s41598-022-18753-0 (PMC9421623; doi:10.1038/s41598-022-18753-0)
Supplement: Supplementary file 1 — Supplementary Figure S1. [file 41598_2022_18753_MOESM1_ESM.docx]

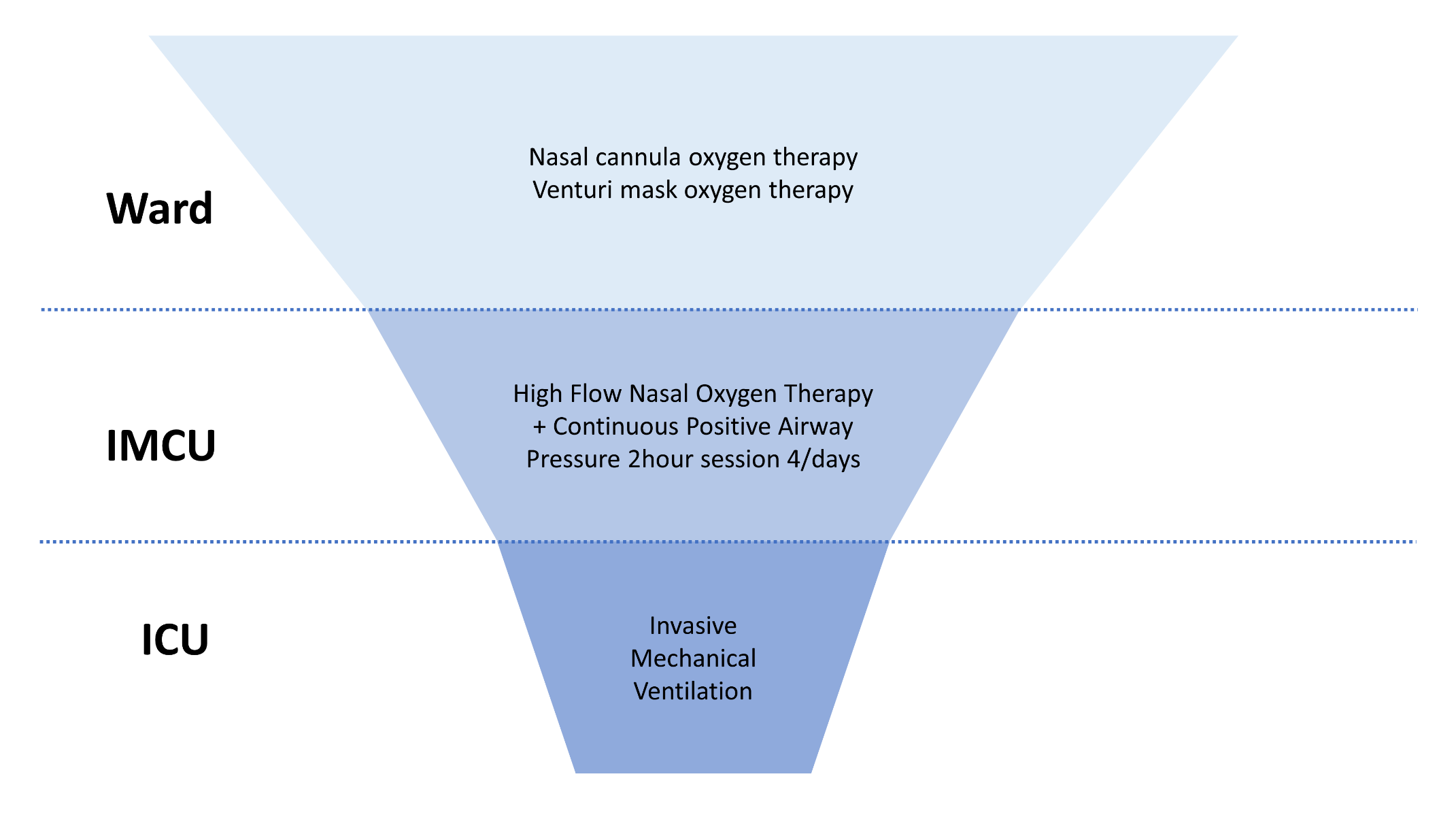


**Figure S1**

Flowchart of oxygen therapy for patient admitted for COVID-19. IMCU: intermediate care unit, ICU: intensive care unit
