# Supplementary material for: Abdominal pain patterns during COVID-19: an observational study
Source: Sci Rep. 2022 Aug 29;12:14677. doi: 10.1038/s41598-022-18753-0 (PMC9421623; doi:10.1038/s41598-022-18753-0)
Supplement: Supplementary file 2 — Supplementary Table S1. [file 41598_2022_18753_MOESM2_ESM.docx]

|  | Univariate logistic regression | | | Multivariate logistic regression | | |
| --- | --- | --- | --- | --- | --- | --- |
|  | **OR** | **95% CI** | **p value** | **OR [Δ]** | **95% CI** | **P value [Δ]** |
| History of abdominal pain or abdominal point tenderness | 1.02 | 0.76-1.38 | 0.887 | 1.19 [-0.05] | 0.85-1.66 | 0.307 [+0.106] |
| History of abdominal pain | 0.97 | 0.70-1.34 | 0.842 | 1.10 [-0.06] | 0.76-1.57 | 0.620 [+0.210] |
| Abdominal point tenderness | 1.20 | 0.85-1.69 | 0.306 | 1.40 [-0.02] | 0.95-2.06 | 0.090 [+0.016] |
| History of right upper region pain | 1.23 | 0.57-2.66 | 0.598 | 1.28 [+0.01] | 0.55-3.02 | 0.566 [-0.014] |
| History of epigastric region pain | 0.64 | 0.33-1.24 | 0.184 | 0.88 [-0.04] | 0.43-1.78 | 0.713 [-0.099] |
| Right upper region tenderness | 2.06 | 1.01-4.23 | **0.047** | 2.80[-0.051] | 1.25-6.28 | **0.012 [+0.002]** |
| Epigastric region tenderness | 0.32 | 0.13-0.77 | **0.011** | 0.38 [-0.04] | 0.15-0.96 | **0.042 [-0.022]** |
| Murphy’s sign | 4.80 | 1.52-15.70 | **0.008** | 6.32 [+0.26] | 1.73-23.09 | **0.005 [0]** |

**Table S1**
