# Supplementary material for: Abdominal pain patterns during COVID-19: an observational study
Source: Sci Rep. 2022 Aug 29;12:14677. doi: 10.1038/s41598-022-18753-0 (PMC9421623; doi:10.1038/s41598-022-18753-0)
Supplement: Supplementary file 3 — Supplementary Table S2. [file 41598_2022_18753_MOESM3_ESM.docx]

|  |  |  | p value, Cochran’s Q |
| --- | --- | --- | --- |
|  | **Upper abdomen pain** | **Lower abdomen pain** |  |
| Dyspnea n (%) | 46/73 (63) | 8/31 (25.8) | <0.001 |
|  | **Upper abdomen tenderness** | **Lower abdomen tenderness** |  |
| Dyspnea n (%) | 43/74 (58.1) | 30/62 (48.4) | 0.010 |

**Tables S2**
