# Supplementary material for: Abdominal pain patterns during COVID-19: an observational study
Source: Sci Rep. 2022 Aug 29;12:14677. doi: 10.1038/s41598-022-18753-0 (PMC9421623; doi:10.1038/s41598-022-18753-0)
Supplement: Supplementary file 4 — Supplementary Table S3. [file 41598_2022_18753_MOESM4_ESM.docx]

|  | Pa0_2_/Fio_2_ <40 [kPa] | | | Abdominal pain & Pa0_2_/Fio_2_<40 [kPa] | | |
| --- | --- | --- | --- | --- | --- | --- |
|  | **OR** | **95% CI** | **p value** | **OR** | **95% CI** | **P value** |
| Mortality \|ICU admission\|IMCU admission | 13.14 | 4.02-43 | **<0.001** | 5.43 | 0.63-47.12 | **0.002** |
| Mortality | 0.73 | 0.24-2.18 | 0.027 | 1.30 | 0.36-4.69 | 0.029 |
| ICU admission | 8.53 | 2.75-26.43 | **<0.001** | 2.80 | 0.76-10.36 | **<0.001** |
| IMCU admission | 3.44 | 1.25-9.44 | 0.059 | 0.98 | 0.34-2.86 | 0.484 |

**Table S3**

Mortality and/or intensive care unit (ICU) admission and/or intermediate care unit (IMCU) admission, mortality alone, ICU alone IMCU alone association with low admission Pa0_2_/Fio_2_ ratio (we use the cutoff of 40kPa or 300mmHg for mild Acute Respiratory Distress Syndrome [The ARDS Definition Task Force. Acute Respiratory Distress Syndrome: The Berlin Definition. JAMA. 2012;307(23):2526–2533. doi:10.1001/jama.2012.5669]) after adjustment for sex, age, abdominal surgery during hospital stay and dyspnea (complete case analyses). OR: odds ratio, CI: confidence interval, kPa: kilo Pascal
