# Supplementary material for: Abdominal pain patterns during COVID-19: an observational study
Source: Sci Rep. 2022 Aug 29;12:14677. doi: 10.1038/s41598-022-18753-0 (PMC9421623; doi:10.1038/s41598-022-18753-0)
Supplement: Supplementary file 5 — Supplementary Table S4. [file 41598_2022_18753_MOESM5_ESM.docx]

|  |  | *n* |
| --- | --- | --- |
| CRP admission [mg/L] median, IQR | 51.7, 18.4-97.5 | 785 |
| CRP max [mg/L] median, IQR | 98.1, 41.7-180.4 | 923 |
| Hemoglobin admission [g/l] mean, SD | 129.4, 21.1 | 809 |
| Hemoglobin min [g/l] mean, SD | 114.6, 21.8 | 949 |
| WBC admission | 7, 3.7 | 802 |
| WBC max [G/l] mean, SD | 9.1, 5.1 | 935 |
| Platelets admission [G/l] mean, SD | 218.6, 98.4 | 808 |
| Platelets min [G/l] mean, SD | 196.3, 81.1 | 946 |

**Table S4**

Laboratory values of included patients. CRPmax: maximal value of the C-reactive protein during the first 30 hospitalization days, Hemoglobin min: minimum value of hemoglobin during the first 30 hospitalization days, IQR: interquartile range, platelets min: minimum value of platelets during the first 30 hospitalization days WBC: white blood cells, WBC max: WBC maximal value during the first 30 hospitalization days.
